# Supplementary material for: MYB-Mediated Regulation of Anthocyanin Biosynthesis
Source: Int J Mol Sci. 2021 Mar 18;22(6):3103. doi: 10.3390/ijms22063103 (PMC8002911; doi:10.3390/ijms22063103)
Supplement: Supplementary file 1 [file ijms-22-03103-s001.pdf]

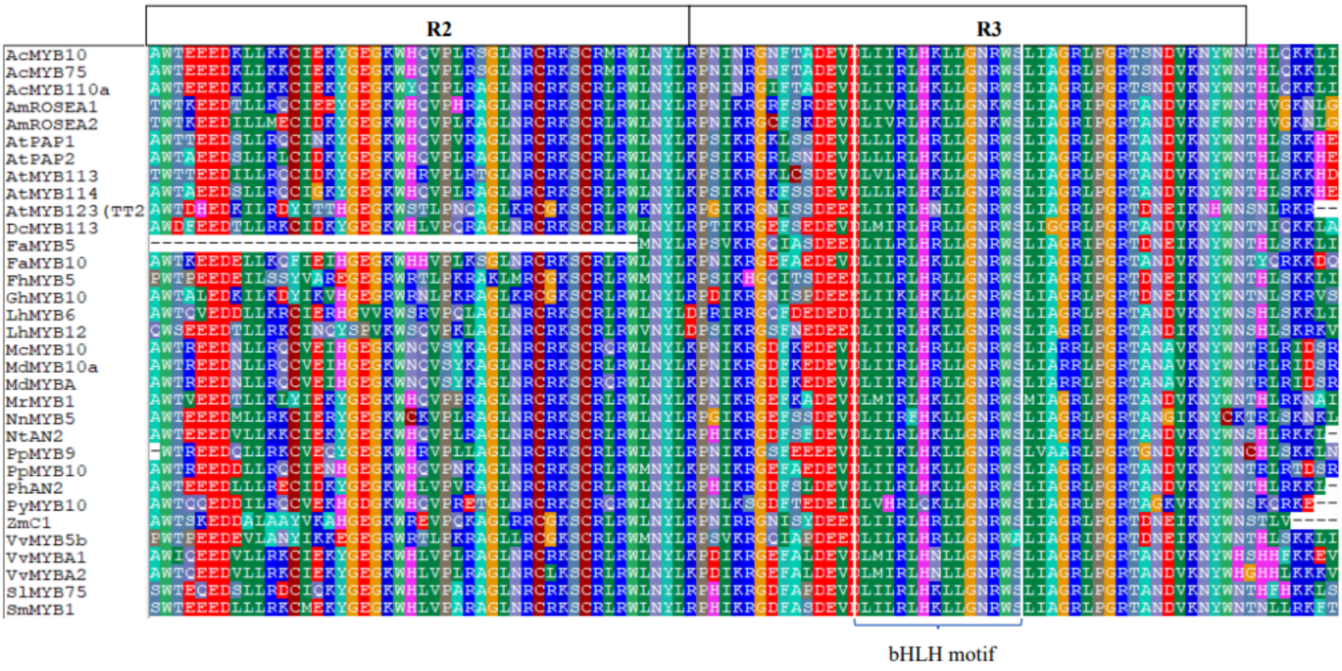

Supplementary figure1. Multiple alignments of the protein sequences MYB-TFs. The R2 and R3 domains are found. The bHLH binding motif is boxed in the R3 domain.
